# Microbial changes in relation to oral mucositis in autologous hematopoietic stem cell transplantation recipients

**DOI:** 10.1038/s41598-019-53073-w

**Published:** 2019-11-15

**Authors:** Alexa M. G. A. Laheij, Judith E. Raber-Durlacher, Renée G. A. Koppelmans, Marie-Charlotte D. N. J. M. Huysmans, Carin Potting, Stephanie J. M. van Leeuwen, Mette D. Hazenberg, Michael T. Brennan, Inger von Bültzingslöwen, Jan-Erik Johansson, Johannes J. de Soet, Thijs M. Haverman, Mark J. Buijs, Bernd W. Brandt, Frederik R. Rozema, Nicole M. A. Blijlevens, Egija Zaura

**Affiliations:** 1 Department of Oral Medicine, Academic Centre for Dentistry Amsterdam, University of Amsterdam and Vrije Universiteit Amsterdam, Amsterdam, The Netherlands; 2 Department of Preventive Dentistry, Academic Centre for Dentistry Amsterdam, University of Amsterdam and Vrije Universiteit Amsterdam, Amsterdam, The Netherlands; 3 Department of Oral and Maxillofacial Surgery, Amsterdam UMC, University of Amsterdam, Amsterdam, the Netherlands; 4 Department of Dentistry, Radboud University Medical Center, Nijmegen, The Netherlands; 5 Department of Hematology, Radboud University Medical Center, Nijmegen, The Netherlands; 6 Department of Hematology, Amsterdam UMC, University of Amsterdam, Amsterdam, The Netherlands; 7 Department of Oral Medicine, Carolinas Medical Center, Charlotte, NC United States of America; 8 Department of Oral Microbiology and Immunology, University of Gothenburg, Gothenburg, Sweden; 9 Department of Hematology and Coagulation, Sahlgrenska University Hospital, Gothenburg, Sweden

**Keywords:** Oral medicine, Oral microbiology, Myeloma

## Abstract

The aim of this prospective, two center study was to investigate the dynamics of the microbial changes in relation to the development of ulcerative oral mucositis in autologous SCT (autoSCT) recipients. Fifty-one patients were diagnosed with multiple myeloma and treated with high-dose melphalan followed by autoSCT. They were evaluated before, three times weekly during hospitalization, and three months after autoSCT. At each time point an oral rinse was collected and the presence or absence of ulcerative oral mucositis (UOM) was scored (WHO scale). Oral microbiome was determined by using 16S rRNA amplicon sequencing and fungal load by qPCR. Twenty patients (39%) developed UOM. The oral microbiome changed significantly after autoSCT and returned to pre-autoSCT composition after three months. However, changes in microbial diversity and similarity were more pronounced and rapid in patients who developed UOM compared to patients who did not. Already before autoSCT, different taxa discriminated between the 2 groups, suggesting microbially-driven risk factors. Samples with high fungal load (>0.1%) had a significantly different microbial profile from samples without fungi. In conclusion, autoSCT induced significant and reversible changes in the oral microbiome, while patients who did not develop ulcerative oral mucositis had a more resilient microbial ecosystem.

## Introduction

Inflammation of the mucosa – mucositis – is a common, dose limiting complication of high dose chemotherapy and radiotherapy. It can affect the mucosa of the whole gastrointestinal tract from the oral cavity to the rectum. Oral mucositis affects about 40–70% of patients treated with myeloablative conditioning chemotherapy for hematopoietic stem cell transplantation (SCT) or other types of high dose chemotherapy^1–4^.

Over the last decades, it became clear that the pathogenesis of mucositis is not limited to the epithelial layer of the mucosa, but also involves submucosal tissues and signaling pathways^5^. Mucositis is initiated by direct and indirect damage to mucosal cells induced by oxidative stress, reactive oxygen species and the immune response following chemo- and/or radiotherapy^6^. Traditionally, five biological phases of mucositis have been described: initiation, up-regulation and activation, signal amplification, ulceration, and healing^5^.

Clinically, mucositis is characterized by redness and/or ulceration of the non-keratinized oral mucosa. Oral mucositis develops on average 5–7 days after the administration of conditioning chemotherapy, peaks at day 12 and lasts about 5 more days^7^. Patients considered oral mucositis as the single most debilitating acute complication of stem cell transplantation^8^. It can be so painful that oral functions such as eating, drinking and speaking, as well as sleeping may become difficult or even impossible^9^. Oral mucositis is associated with prolonged hospitalization, polypharmacy (e.g.antibiotic and opioid use), parenteral nutrition,and higher costs of care^4,10^. In 2018, 23 000 transplants were performed in the USA^11^. Ulceration of the oral mucosa leads to a breach in the natural defense barrier and provides oral microorganisms and inflammatory cytokines a portal of entry into underlying tissues and the systemic blood supply^5,12^. Particularly during neutropenia, ulcerative oral mucositis (UOM) may be associated with fever^13^, and bacteremia may lead to life threatening systemic infections.

Currently, there is a surge of interest in the role of the microbiome in health and disease^14,15^. When it comes to mucositis, it was thought that microorganisms arbitrarily colonize the ulcerative lesions thereby intensifying inflammation^5^. However, recent studies suggest that the role of the microbiome in both intestinal and oral mucositis may be more extensive. Dysbiosis of the microbiome is reported following anticancer agents and coinciding with clinical manifestations of mucositis^16–21^. Some studies report differences in the microbiome in mucositis and non-mucositis patients^3,20,22,23^. Mechanistically, it is suggested that the microbiome influences mucositis by acting on the immune response via TLR^21^, NFκB, or mitogen-activated protein kinase (MAPK) signaling^17^. Alternatively, oral inflammatory processes such as periodontitis that are modulated by the microbiome, may prime the patient for these inflammatory processes^24^.

To date, over 700 bacterial species have been detected in the oral cavity^25^. The oral mycobiome seems to consist of at least 31 genera^26^. Studies suggest that certain bacterial and fungal species are associated with the presence of ulcerative oral mucositis in SCT recipients^3,27^. For instance, microorganisms that are traditionally associated with periodontitis, e.g. *Porphyromonas gingivalis*^3^, but also *Enterococcus* species^22^ and *Candida* species are associated with oral mucositis after SCT^3,22,23^.

However, well-designed prospective studies in SCT patients using state-of- the-art techniques are lacking, while current studies largely have a cross-sectional design. Therefore, the aim of this study was to longitudinally assess the dynamic changes in the oral microbiome relative to the development of ulcerative oral mucositis in autologous SCT (autoSCT) recipients. Furthermore, we explored whether the presence of specific bacterial species could be predictive for ulcerative oral mucositis. We also aimed to identify changes in fungal load after autoSCT, and to relate any changes to the development of ulcerative oral mucositis.

## Results

### Clinical outcomes of the study

In total, 51 multiple myeloma patients were included in the study. The mean age was 57. 4 ± 7.3 years; 28 participants were male (55%) and 23 were female (45%). After autoSCT, 28 patients had a maximum WHO oral mucositis score of 0, three had a maximum score of 1, 13 had a maximum score of 2, 5 had a maximum score of 3, and 2 patients had a maximum score of 4. Since ulcerative stages of oral mucositis (WHO ≥ 2) are clinically most relevant, patients were allocated into two groups: patients that developed ulcerative oral mucositis (UOM patients, WHO ≥ 2, n = 20), and patients not developing oral mucositis (WHO = 0) or only mild, non-ulcerative oral mucositis (WHO = 1) (N-UOM patients, n = 31). Ulcerative oral mucositis usually started one week after autoSCT and mostly lasted between two and 14 days. None of the patients had oral mucositis three months after autoSCT.

On average, patients were admitted to the hospital for 21.9 (±7.1) days and used ciprofloxacin for 15.8 (±5.2) days. Patients with UOM had longer hospitalizations compared to N-UOM patients (24.5 (±10.4) vs 20.2 (±2.8) days, p = 0.005). There was no difference between the groups with respect to the number of days of ciprofloxacin use (p > 0.05).

### Overall sequencing output

In total, 86% of reads were merged and passed quality filtering, with a mean of 14867 reads (SD: 4156, min: 5666, max: 25430) per sample. The reads were grouped in 483 minimum entropy decomposition nodes (MEDs). After removing the nodes that originated from controls, 478 MEDs remained in the dataset. These were classified in 62 genera or higher taxa. The most abundant genus was *Streptococcus* (24% of total reads), followed by *Prevotella* (20%), *Veillonella* (20%), *Rothia* (6%), *Actinomyces* (5%), *Lactobacillus* (4.5%) and *Staphylococcus* (2.5%).

### Oral microbiome and ulcerative oral mucositis

**Figure 1:**
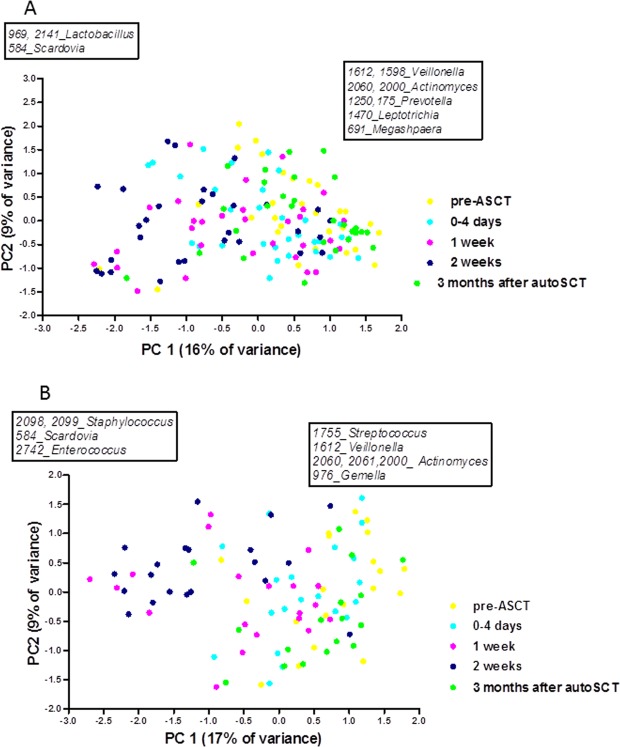
PCA on oral microbiome profiles by time point, (**A**) cases without ulcerative oral mucositis and (**B**) cases who developed ulcerative oral mucositis. Boxes indicate MEDs that contributed most to PC1. Left box: more abundant at low values of PC1, right box: more abundant at high values of PC1.

First, we ordinated the microbiome profiles by principal component analysis (PCA). Independent of the development of ulcerative oral mucositis, the oral microbiomes changed significantly over time (p = 0.0001, PERMANOVA, Fig. 1A,B). In both groups, the microbial composition of samples collected before autoSCT (pre-autoSCT) differed from those at one and two weeks after autoSCT. However, in the UOM group, the difference after one week was more pronounced (F = 3.04, p = 0.001) than in the N-UOM group (F = 2.4, p = 0.04). At two weeks, samples of both groups differed from the respective pre-autoSCT samples at the same significance (p = 0.001). In both groups, three months after autoSCT the oral microbiome profiles had returned to the pre-autoSCT composition (p > 0.05).

Next, based on the PCA loadings, we assessed which taxa were responsible for the observed microbial shifts over time. In the N-UOM group, pre- and three months post-autoSCT microbial communities were high in MEDs classified as *Veillonella atypica/dispar* (MED1612), *Actinomyces* sp. OT172 (MED2060), *Actinomyces graevenitzii* (MED2000), genus *Prevotella* (MED1250), genus *Leptotrichia* (MED1470), *Megasphaera micronuciformis* (MED691), and *Veillonella* sp. OT917 (MED1598), while samples at one and two weeks after stem cell infusion were enriched in MEDs classified as genus *Lactobacillus* (MED2141), *Lactobacillus fermentum* (MED969) and *Scardovia wiggsiae* (MED584).

In the UOM group, the samples from before and three months after autoSCT had a higher proportion of MEDs classified as *Streptococcus australis*/*parasanguinis*/OT057/OT066 (MED1755), *Veillonella atypica/dispar* (MED1612), genus *Actinomyces* (MED2061), *Actinomyces* sp. OT172 (MED2060), *Actinomyces graevenitzii* (MED2000) and *Gemella haemolysans/morbillorum/sanguinis* (MED976), while one and two weeks after stem cell infusion these samples showed increased abundance of MEDs classified as *Staphylococcus aureus/caprae/epidermidis/warneri* (MED2099, MED2098), *Scardovia wiggsiae* (MED584), and *Enterococcus faecalis* (MED2742). No differences in microbial profile were present between the different transplant centers at any time point (data not shown).

Thereafter, we assessed the magnitude of changes in the microbial profiles over time, in relation to the samples collected before autoSCT (Bray-Curtis Similarity Index, Fig S1). The similarity of the pairs of samples (each consecutive time point compared to pre-autoSCT samples) decreased significantly after autoSCT in both groups (GLM RN test, p < 0.001). However, the decrease in similarity appeared significantly sooner, was more pronounced, and lasted longer in the UOM group compared to the N-UOM group.

**Figure 2:**
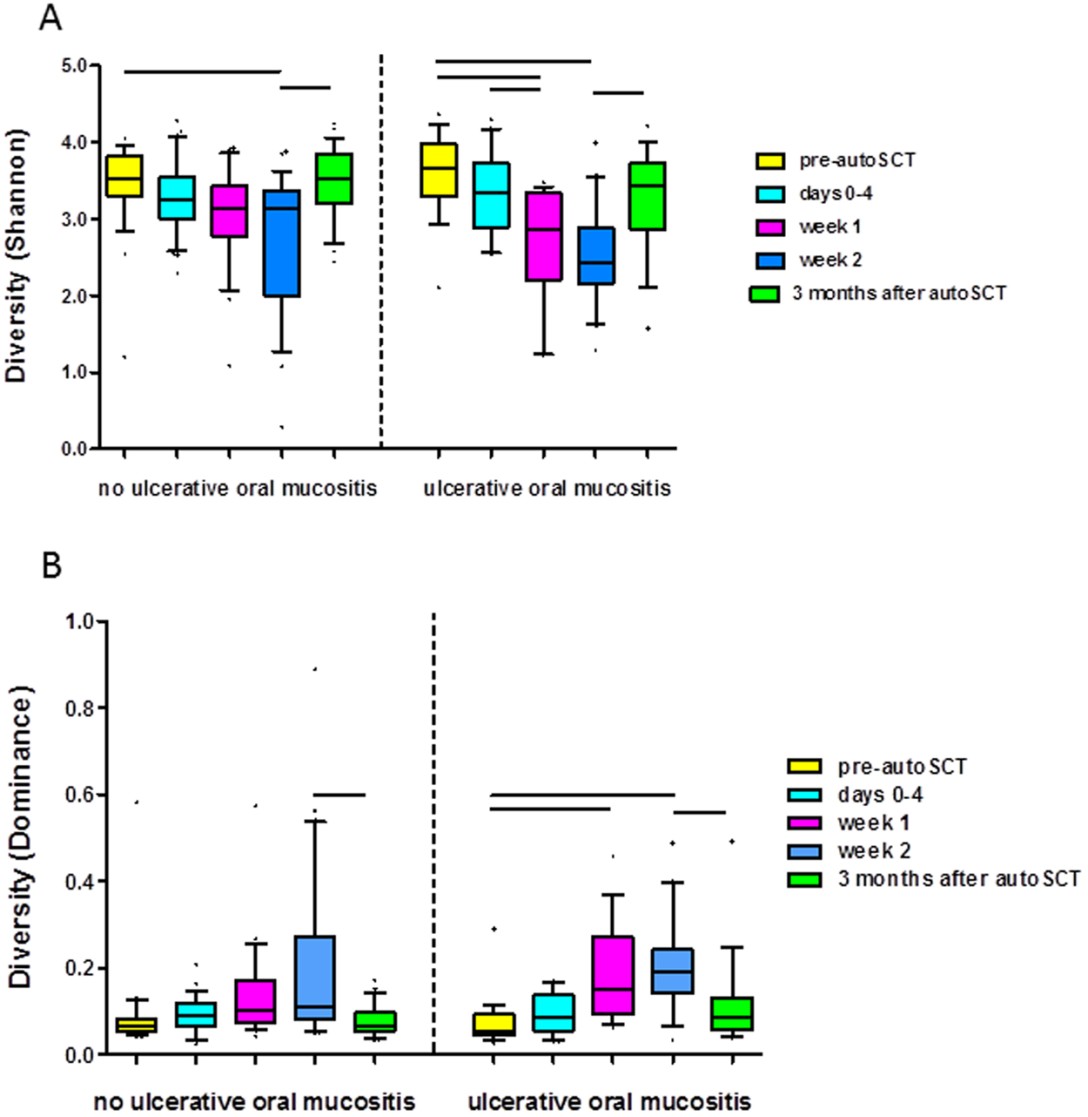
Diversity of oral microbiome profiles per group by time point, (**A**) Shannon index, (**B**) Dominance index. Lines connect significantly different time points (GLM RM test, p < 0.01).

Next, we assessed changes in the diversity of the oral microbiomes of the autoSCT patients per group (Fig. 2). The Shannon diversity index decreased in N-UOM samples at two weeks after stem cell infusion (GLM RM test, p < 0.01, Fig. 2A), while no significant changes in Dominance index were observed in these patients (Fig. 2B). In contrast, samples from UOM patients showed a significant reduction in Shannon diversity index (Fig. 2A), and a significantly increased dominance index (Fig. 2B) already at week 1. At three months after autoSCT, the diversity had significantly increased again in both groups and was comparable to pre-autoSCT. When the microbiome profiles of the two groups were assessed per individual time point, no significant differences were found (PERMANOVA, p > 0.05) (data not shown).

**Figure 3:**
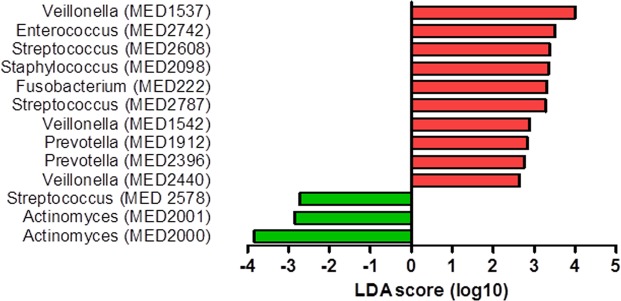
MEDs that significantly discriminated the two groups –patients that did not develop ulcerative oral mucositis (green) and patients that did develop ulcerative oral mucositis (red) before autoSCT (biomarker identification tool LEfSe, p < 0.05, LDA > 2).

### Individual microbial taxa in relation to ulcerative oral mucositis

To assess which microbial taxa (MEDs) were associated with the development of ulcerative oral mucositis, the biomarker identification tool LEfSe was used. Before autoSCT, 13 MEDs discriminated significantly between the two groups (LEfSe, p < 0.05, LDA > 2, Fig. 3). Pre-autoSCT samples from N-UOM patients contained a higher proportion of reads classified as *Actinomyces graevenitzii* (MED2000, MED2001) and *Streptococcus constellatus* (MED2578), while samples from UOM patients showed a higher proportion of reads classified as genus *Veillonella* (MED1537, MED1542, MED2440), *Enterococcus faecalis* (MED2742), genus *Streptococcus* (MED2608, MED2787), *Staphylococcus* spp. (MED 2098), genus *Fusobacterium* (MED222), *Prevotella oris* (MED1912) and *Prevotella veroralis* (MED2396).

### Fungal load

**Figure 4:**
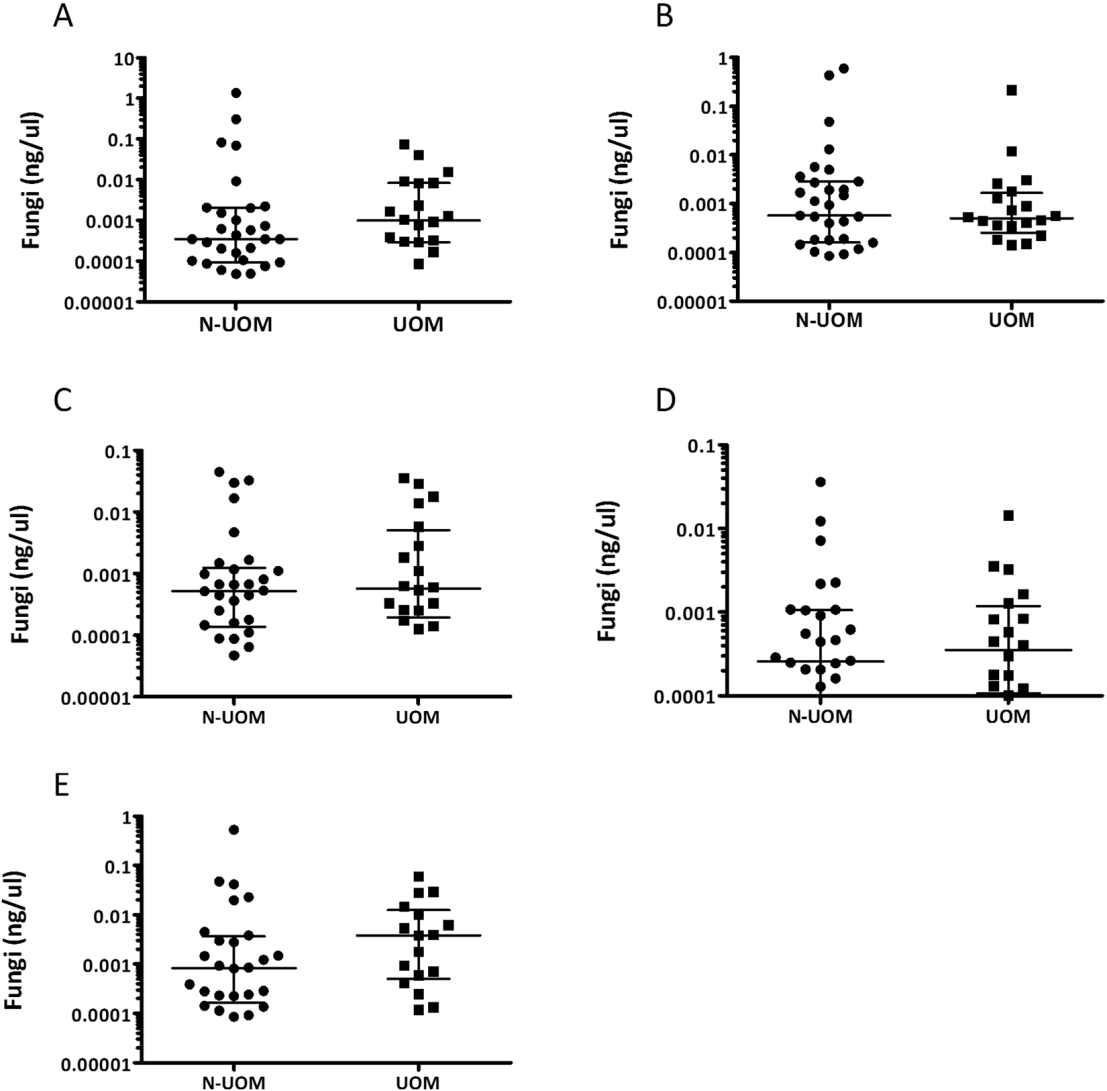
Relative abundance of fungi in the samples of patients who did and did not develop ulcerative oral mucositis (**A**) before autoSCT, (**B**) 0–4 days after autoSCT, (**C**) one week, (**D**) two weeks, (**E**) three months after autoSCT. Bar connects significant differences (Mann Whitney U test). Fungi were detected using primers from Vollmer *et al*.^26^.

From all samples, 91% contained detectable fungal DNA. The median fungal load as a proportion of the bacterial load (16S rDNA) was 0.02% (range 0–25.2%) per sample. Most samples (75%) contained a low (<0.1%) fungal load. Four samples had a very high (10–25%) proportion of fungi. Before autoSCT and in the first weeks after autoSCT, there was no difference in proportion of fungi between the groups, while at three months post-autoSCT, the UOM patients had a significantly higher proportion of fungi in their oral microbiomes compared to N-UOM patients (p = 0.036, Mann-Whitney test, Fig. 4). There was no association between the fungal load and the age of the patients nor between the fungal load and the number of natural teeth the patients had in their oral cavity (p > 0.05). There was also no significant difference in fungal load at any time and smoking habits (p > 0.05).

**Figure 5:**
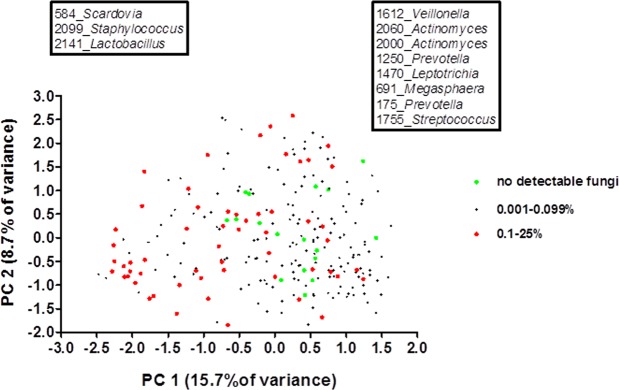
PCA of salivary microbial profiles, colored by fungal load relative to bacterial DNA (16S rDNA). Microbiome of samples with 0.1% fungi or higher (red) differed significantly from those with no fungi (green) (F = 3.106, p < 0.05, PERMANOVA). The boxes indicate the MEDs that were associated with high fungal load (left box) or low fungal load (right box), based on PC1 loadings.

Samples with high fungal load (>0.1%) had a significantly different microbial profile compared to samples with less or no fungi present (Fig. 5). Based on PCA loadings, MEDs classified as *Scardovia*, *Staphylococcus* and *Lactobacillus* were associated with a high fungal load in the oral cavity. Despite differences in the use of antifungal prophylaxis between the two transplant centers, there was no significant difference in the proportion of fungi between the study centers at any time point (Mann-Whitney U test, p > 0.05).

## Materials and Methods

**Figure 6:**
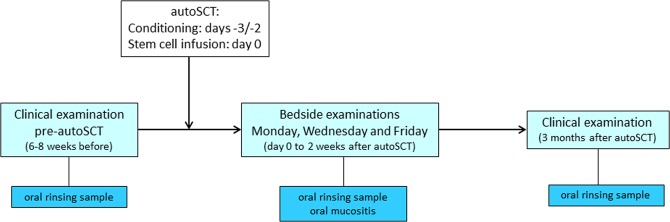
Study flow diagram.

An observational, prospective, longitudinal study on the oral microbiome and ulcerative oral mucositis was conducted during October 2015 and March 2017 in 51 patients who received an autologous hematopoietic stem cell transplantation (autoSCT) for multiple myeloma at the Amsterdam University Medical Centers location AMC (N = 27) or at the Radboud University Medical Center Nijmegen (N = 24). This study was performed as a sub-study of a large multinational study on the impact of oral side effects from conditioning therapy before SCT (Orastem)^28^. For a schematic representation of the study flow see Fig. 6.

### Ethics statement

The study was approved by the Medical Research Ethical Committee from Amsterdam University Medical Centers location AMC and the Radboud University Medical Center Nijmegen (NL52117.018.15), trial number: NL5645. The methods were carried out in accordance with the relevant guidelines and regulations. Written informed consent was obtained from all patients. Seventy adult patients were asked to participate, 15 patients refused to participate, two patients could not be included because of logistics, whereas two included patients did not receive an autoSCT and were excluded from this study.

### Patients and sampling

Patients did not receive antibiotic treatment prior to autoSCT, but received antimicrobial prophylaxis starting at the time of autoSCT. In the Amsterdam UMC, prophylaxis consisted of orally administered ciprofloxacin, feneticillin and fluconazole, whereas in the Radboud UMC, prophylaxis consisted of oral ciprofloxacin only. Following autoSCT, therapeutic antibiotics and/or antimycotics were prescribed to all patients, when indicated. All patients received additional antibiotic therapy and 31 patients received systemic or non-systemic antimycotics. Additional antibiotic treatment consisted of cephalosporins, macrolides, glycopeptides, nitroimidazole, polymyxins, penicillins or sulfonamides. Conditioning consisted of melphalan 200 mg/m^2^. Forty patients completed the hospital protocol regarding oral cryotherapy^29^.

At a median of 36 days (range −124 to −4) before autoSCT, all participants received a full dental examination, including radiographs, caries score (ICDAS) and a full periodontal evaluation by calibrated dentists. Evident oral foci (e.g., semi-impacted third molars, deep caries, periapical lesions, deep periodontal pockets) were eliminated as much as possible before autoSCT. During hospitalization, institutional oral care protocols (not including chlorhexidine rinses) were followed.

For oral sampling participants were asked to rinse the oral cavity thoroughly for 20–30 seconds with 10 ml of sterile 0.9% saline solution. The solution was collected in a sterile tube, kept on ice and centrifuged at 4500 g for 7 min within two hours. Pellets were resuspended in 1 ml sterile PBS and stored at −80 °C until analysis. In some cases, oral sampling could not be performed due to pain and/or discomfort.

During the neutropenic phase, oral mucositis was scored according to the criteria of the World Health Organization^30^ at eight anatomical sites (labial and buccal mucosa, floor of the mouth, lateral and ventral tongue, and soft palate) by calibrated researchers. The scores were recalculated into no ulcerative or only mild non-ulcerative oral mucositis (N-UOM; WHO ≤ 1) versus ulcerative oral mucositis (UOM; WHO ≥ 2).

### Sample selection, preparation, 16S rDNA amplicon sequencing and data processing

All pre-autoSCT oral samples were analyzed. During hospitalization, the collection of oral rinsing samples was attempted three times a week (on Monday, Wednesday and Friday), however, sometimes patients were too ill to provide a sample. Not all samples were sequenced. From all patients one sample per week was selected. A sample was collected at the day of the stem cell infusion (day 0) or as soon as possible thereafter. In N-UOM patients, samples collected within a regular interval (at one and two weeks after stem cell infusion) were selected. In UOM patients, the same regular intervals were chosen, including that particular sample which coincided with the onset of clinical presentation of ulceration.

Oral rinse samples were thawed and pelleted by centrifugation, the supernatant was removed and the pellet was resuspended in 100 µL TE buffer and added to an assigned well in a deepwell plate containing 100 μL lysis buffer (Mag Mini DNA Isolation Kit, LGC, Hoddesdon, UK), 250 μL zirconium beads (0.1 mm; BioSpec products, Bartlesville, OK, USA) and 200 μL phenol saturated with Tris-HCl (pH 8.0; Carl Roth, Germany). Samples were placed in a Mini-BeadBeater-96 (BioSpec products, Bartlesville, OK, USA) for two min at 2100 oscillations/min. DNA was extracted using the Mag Mini DNA Isolation Kit^31^. Bacterial DNA concentration was determined by quantitative PCR, with universal primers specific to the bacterial 16S rRNA gene according to Ciric *et al*.^32^.

Amplicon sequencing of V4 region of 16S rDNA was performed according to Koopman *et al*.^33^ with the exception that the samples were sequenced two times 250 cycles at the Tumor Genome Analysis Core (www.tcga.nl, CCA, Amsterdam UMC, Vrije Universiteit Amsterdam, the Netherlands) and that the flowcell was loaded with 7 pmol including 40% PHix.

The reads were merged and quality filtered^33^, after which the sequences were decomposed into nodes using Minimum Entropy Decomposition (v.2.1)^34^, further called MEDs. In detail, the merged and quality-filtered sequences were processed as follows: after de-replication (excl. singletons), chimeric sequences were removed using USEARCH v8.0.1623^35^ (uchime_denovo). The resulting non-chimeric sequences were subsampled to 4500 reads/sample, while retaining samples with less than 4500 reads (default in MED subsampling). Next, the sequences were decomposed into MED nodes using a min. substantive abundance of 100, relocation of outliers, and otherwise default parameters. Taxonomy was assigned to the node representatives using the RDP classifier^36^ (via QIIME v.1.8.0^37^) with a minimum confidence of 0.8 and the Human Oral Microbiome Database (HOMD)^25^. The HOMD-aligned sequence set (v. 14.51) was first trimmed to the V4 region, after which it was converted to a non-redundant set of gap-free sequences to retrain the RDP classifier.

Of the 249 sequenced samples, four were lost due to a too low DNA yield or less than 4500 reads after sequencing. Finally, 245 samples were used for analyses. For 43 patients there were five samples available, while for six patients - four and for two patients – three samples were available for data analyses.

### Fungal analyses

After DNA extraction, the fungal load of the samples was determined using quantitative PCR, as described previously^38^. The fungal load was calculated as the relative percentage of fungal DNA to the amount of bacterial DNA.

### Statistical analysis

Differences were calculated using the t-test or Mann-Whitney test, differences between averages for longitudinal data were calculated using General Linear Model (GLM) or Generalized Estimating Equation (GEE analysis). Correlations were calculated with the Pearson (scale data) or with the Spearman (ordinal data) correlation (SPSS, version 26). Principal component analysis (PCA), permutational multivariate analysis of variance (PERMANOVA) using the Bray-Curtis similarity distance, the Shannon diversity index and Dominance index were calculated using PAST software v. 3.20^39^. The LDA effective size (LEfSe) biomarker discovery tool was used with the ‘one against all’ strategy for multiclass analysis and logarithmic LDA score threshold of 2, p < 0.05^40^. A p-value < 0.05 was considered to be statistically significant.

## Discussion

In this prospective longitudinal study, we assessed the dynamics of the relationship between the oral microbiome and the development of ulcerative oral mucositis (UOM) in patients who received high dose chemotherapy prior to autologous stem cell transplant (autoSCT). High-dose hemotherapy for autoSCT induced significant, but reversible changes in the oral microbiome. Interestingly, patients who did not develop ulcerative oral mucositis exhibited a more resilient microbiome and distinct microbial taxa before autoSCT that differentiated them from patients who did develop ulcerative oral mucositis (N-UOM).

So far, the dynamics of specific bacterial and fungal species in relation to oral mucosa after SCT has been addressed using either culturing or PCR-based approaches using mostly cross-sectional study designs^3,22,23,27,41^. The only published study assessing oral bacterial profiles using state of the art, open end techniques in comparable way to our study, used a different study population – active disease nasopharyngeal carcinoma patients, all receiving radiotherapy, with or without additional chemotherapy^20^. Similar to our findings, they also observed a significant decrease in microbial diversity after the cancer treatment. A decrease in microbial diversity in the oral cavity is, besides being related to oral mucositis, also associated with other, more common oral diseases, like dental caries^42,43^.

The changes in the microbiome occurred in all patients receiving autoSCT, however, these changes were less pronounced and slower in patients who did not develop ulcerative oral mucositis, suggesting a more resilient oral microbiome. Alternatively, this may reflect a less severe disease phenotype with cause and consequence remaining difficult to dissect^44^. Nonetheless, distinct differences were observed in the timing and severity of dysbiosis in patients without clinically observed ulcerative oral mucositis, suggesting the microbiome may be a possible biomarker of mucositis severity.

Three months after autoSCT, the oral microbial composition, diversity and similarity returned to pre-autoSCT levels in both groups. The mechanisms of resilience of microbial communities are still poorly understood. These may be related to bacterial inter-individual interactions such as nutritional factors, physical interactions, antagonistic interactions, cell-cell signaling and gene transfer^19,45^ and/or to host factors such as the status of the immune system or the integrity of the underlying substratum – oral mucosa^46^. In our study both, bacterial and host factors may have affected the observed difference in resilience between UOM and N-UOM patients. After all, there were different bacterial communities present already before autoSCT and at the occurrence of ulcerative oral mucositis in both groups. There may have also been differences in the immune system and/or the microcirculation of the mucosa. All these factors may have influenced the resilience of the microbiota, yet further studies are necessary to unravel the exact mechanisms of the host microbe interactions involved in this process.

*Scardovia* spp and *Lactobacillus* spp were associated with preserving oral mucosal integrity after SCT. *L*. *brevis* has been reported to reduce UOM in head and neck cancer patients^47^, and *L*. *acidophillus* reduced the severity of 5-FU induced gastrointestinal mucositis^48^. *Scardovia* spp and *Lactobacillus* spp are Gram-positive bacteria that are able to ferment sugars into lactic acid and their increase is usually associated with dental caries^45,49^, suggesting acidification of the oral ecosystem. In addition, pre-autoSCT oral microbiome of patients who maintained an intact oral mucosa following autoSCT, contained a higher proportion of *Streptococcus* and *Actinomyces* already before the therapy. These two bacterial genera are Gram-positive facultative anaerobes. They are early colonizers of dental hard tissues and are generally associated with oral health^50^.

On the other hand, *Staphylococcus* spp and *Enterococcus* spp were associated with the presence of ulcerative oral mucositis. These are Gram-positive facultative anaerobic, opportunistic bacteria that are commonly associated with non-oral infections and disease, including gastrointestinal mucositis^51–53^. Nevertheless, these opportunistic bacteria^54,55^ may be present in immunocompromised cancer patients^56^. Osakabe *et al*. recently also found an association between *Enterococcus* spp and oral mucositis in SCT patients^22^. Moreover, *Staphylococcus* spp were cultured during episodes of bacteremia in SCT recipients with oral mucositis^57,58^. Furthermore, Gram-negative anaerobic bacterial taxa that are traditionally associated with periodontitis: *Fusobacterium* spp and *Prevotella* spp^59^, and opportunistic pathogens: *Staphylococcus* spp and *Enterococcus* spp were among indicators for UOM development. Whether these bacterial taxa have a role in the dynamics of the host microbe interaction in oral mucositis needs to be confirmed in future studies. However, these preliminary data suggest the presence of microbial-based risk factors in ulcerative oral mucositis and parallel emerging phenomena were described in gastrointestinal mucositis^19^.

Zhu *et al*. found that patients who eventually developed severe oral mucositis transiently harbored a notably higher proportion of *Actinobacillus* spp. during a mild phase of oral mucositis^20^. In our study only a very small proportion of all reads were *Actinobacillus* spp, which could be due to differences in study population and cancer treatment. In addition, all our patients all received antibiotics, while in their study the use of antibiotics in the two weeks before radiotherapy was an exclusion criterium.

Nearly all oral samples were fungi-positive. We observed a significant difference in fungal load between the patients who developed ulcerative oral mucositis after treatment and those who did not only three months after autoSCT. Previous studies report an association between *Candida* spp and oral mucositis^3,22,23^. Our current study and a study on healthy Dutch elderly found a relationship between the composition of the oral microbiome and fungal load^60^. Fungi flourish in acidic conditions and acidogenic species like *Scardovia* and lactobacilli can provide such an environment. Increase of fungal load only after three months suggests that fungi have responded to the ecological changes in the oral environment directly after the therapy but might not be directly involved in the pathogenesis of oral ulcerative mucositis. The role of the mycobiome (the entire fungal community) in this dynamic interaction is however not known and should be addressed in future studies.

Although the course and onset of oral mucositis is fairly predictable^7^, there were individual differences in onset, duration and peak mucositis scores that may be explained by factors that we could not correct for, such as genetic differences. Patients were not chemo naïve, which might have influenced the oral microbiome before SCT. And the use of antimicrobials after autoSCT might have had an influence on the oral microbiome. On the other hand, it has been shown that antibiotics have a slight and only transient effect on the oral microbiome compared to the gut microbiome^61^. It would have been very informative to assess the effects of individual antimicrobial regimens used, however for that purpose much larger number of patients than in the current study would be necessary.

There is a debate on whether next to treatment-related and patient-related factors^44^, the oral microbiota contribute to the pathogenesis of oral mucositis, or only play a modulating role. There is growing evidence that the correct functioning of human cells and organs is co-depending on the host-bacteria interaction. The oral cavity is heavily loaded with micro-organisms that are in constant contact with the oral soft tissues via (non)specific receptors and there is considerable cross-talk^62^. We and others found an association between oral mucositis and the oral microbiome^3,20,22,23^. Several research groups tried to disentangle the specific mechanisms by which oral bacteria and fungi may play a role in oral mucositis^17,21,24,63,64^. However, the nature of the host-microbe interactions in this clinical phenomenon is still undisclosed. Further work remains to appropriately dissect the causative role of host-microbe interactions in mucositis development. Pre-clinical work into host microbe interaction pathways, using 2D and 3D models for oral mucosa and oral microorganisms^65^ identified from clinical studies such as the present study, will forward our understanding. In turn, the obtained results should be validated in future clinical trials on a larger group of patients.

In conclusion, in this prospective longitudinal two-center study, we demonstrated that preparative conditioning regimes for autoSCT induced significant though reversible changes in the oral microbiome irrespective of the development of ulcerative oral mucositis. However, patients who did not develop ulcerative oral mucositis showed a more resilient oral microbial ecosystem than those who did develop ulcerative oral mucositis. Additionally, we identified a higher abundance of mucositis-associated microbial taxa present already before transplantation, suggesting microbial involvement in the pathogenesis of ulcerative oral mucositis and a potential for identifying prognostic biomarkers and subsequently future therapeutic options.

## Data Availability

The datasets generated during and/or analyzed during the current study are available from the corresponding author on reasonable request.
